# Hexamethyldisiloxane Removal from Biogas Using a Fe_3_O_4_–Urea-Modified Three-Dimensional Graphene Aerogel

**DOI:** 10.3390/molecules28186622

**Published:** 2023-09-14

**Authors:** Siqi Lv, Xifeng Hou, Yanhui Zheng, Zichuan Ma

**Affiliations:** 1 Hebei Key Laboratory of Inorganic Nano-Materials, College of Chemistry and Material Sciences, Hebei Normal University, Shijiazhuang 050024, China; lvsq@stu.hebtu.edu.cn; 2 Hebei Key Laboratory of Animal Physiology, Biochemistry and Molecular Biology, College of Life Sciences, Hebei Normal University, Shijiazhuang 050024, China; houxf@stu.hebtu.edu.cn; 3 College of Chemical Technology, Shijiazhuang University, Shijiazhuang 050035, China

**Keywords:** adsorption, hexamethyldisiloxane, reduced graphene oxide aerogel, Fe_3_O_4_-modified, hydrothermo–chemical reduction method

## Abstract

Volatile methyl siloxanes (VMS), which are considered to be the most troublesome impurities in current biogas-cleaning technologies, need to be removed. In this study, we fabricated a series of Fe_3_O_4_–urea-modified reduced graphene-oxide aerogels (Fe_3_O_4_–urea–rGOAs) by using industrial-grade graphene oxide as the raw material. A fixed-bed dynamic adsorption setup was built, and the adsorption properties of the Fe_3_O_4_–urea–rGOAs for hexamethyldisiloxane (L2, as a VMS model pollutant) were studied. The properties of the as-prepared samples were investigated by employing various characterization techniques (SEM, TEM, FTIR, XRD, Raman spectroscopy, and N_2_ adsorption/desorption techniques). The results showed that the Fe_3_O_4_–urea–rGOA–0.4 had a high specific surface area (188 m^2^ g^−1^), large porous texture (0.77 cm^3^ g^−1^), and the theoretical maximum adsorption capacity for L2 (146.5 mg g^−1^). The adsorption capacity considerably increased with a decrease in the bed temperature of the adsorbents, as well as with an increase in the inlet concentration of L2. More importantly, the spent Fe_3_O_4_–urea–rGOA adsorbent could be readily regenerated and showed an excellent adsorption performance. Thus, the proposed Fe_3_O_4_–urea–rGOAs are promising adsorbents for removing the VMS in biogas.

## 1. Introduction

Biogas produced via the anaerobic digestion of organic matter in landfill and sewage plants is a promising alternative to fossil-fuel-based energy [1]. The primary components of biogas are 50–70% CH_4_ and 30–50% CO_2_, and the minor components consist of NH_3_, H_2_S, halogenated compounds, and volatile methyl siloxanes (VMS) [2,3,4]; in particular, VMSs are considered to be the most troublesome in current biogas-cleaning and -upgrading technologies [5]. In recent years, however, VMSs have become widespread in different types of biogas due to the extensive use of polydimethicone in the formulation of personal care products, industrial lubricants, glues, paints, and detergents [6]. The presence of VMSs will decrease the practical applicability of biogas; this occurs because they will be converted into microcrystalline silica during combustion, thus damaging engine devices (i.e., pistons, cylinders, and valves) and inhibiting heat conduction as well as lubrication [6,7]. Thus, the VMS must be removed from biogas prior to use.

Cryogenic condensation, biological technology, catalytic processes, membrane separation, absorption, and adsorption are the most common approaches used to remove the VMS from biogas [6,7,8,9,10,11,12,13,14]. Among the reported methods for the removal of VMS, adsorption has been found to be one of the most effective due to its high efficiency, facile operation, and strong economic feasibility [15,16]. Several adsorbents have been commercially used or explored under development, including activated carbons, silica gels, alumina, molecular sieves, and polymer resins, etc. [12,17,18,19,20,21,22]. While these adsorbents have many attractive properties, such as being simple to use, a high adsorption capacity, and a low cost, their poor cycle performances have limited their commercial applications [23]. Therefore, the exploration of novel material materials as VMS adsorbents with a high adsorption potential and excellent recycling performance is quite necessary.

A reduced graphene oxide aerogel (rGOA) with a three-dimensional porous network structure is formed by the cross-linked stacking of graphene sheets. Zheng et al. [24,25] prepared a series of micro/narrow mesoporous reduced graphene oxide aerogels under hydrothermal conditions by using VC and amine as reducing agents. These aerogels exhibited a good adsorption performance for L2, as well as an excellent cycling stability [24,25]; however, the increase in the specific surface area of the rGOA and the regulation of the pore size were restricted. Therefore, finding new ways of improving the texture properties of rGOAs has continued to attract attention. Recently, many researchers have revealed that a metal oxide-modified rGOA can exhibit good texture features (such as porosity and specific surface area) due to the synergistic effect between the interconnected three-dimensional pores of the rGOA and the rich porous structure of the metal oxide [26,27]. Fe_3_O_4_ has become an ideal metal oxide for the preparation of a modified rGOA due to its advantages of being widely sourced, environmentally friendly, and low cost [28]. Li et al. [29] prepared Fe_3_O_4_–rGOA via a one-step chemical reduction method, making the microwave absorption performance of the composite material significantly higher than that of Fe_3_O_4_ and reduced graphene alone. Vinoshkumar et al. [30] prepared Fe_3_O_4_–rGOA via a hydrothermal method, which is an effective photocatalytic material and has good degradation activity for methylene blue dyes. As of now, the aerogels based on reduced graphene oxide reported in the literature are applied in many fields, such as catalysis [31], conduction [32], gas purification [33,34], electromagnetic wave absorbing [35,36,37], and the adsorption of dyes in water [29,30]. Remarkably, we provide a new aerogel modification method for the removal of VMS. Based on the urea reduced graphene oxide aerogel prepared by Zheng et al. [24], we introduced Fe_3_O_4_ in order to obtain a larger specific surface area and more suitable pore structure for L2 adsorption. According to the structure–activity relationship, the adsorption mechanism was further explored.

To identify the good adsorption performance of Fe_3_O_4_–urea–rGOA for VMS, a series of Fe_3_O_4_–urea–rGOAs was produced with industrial-grade graphene oxide (IGGO) as the raw material, Fe(NO_3_)_3_·9H_2_O as the metal oxide precursor, and urea as the reducing agent via a one-step hydrothermal method. The synthesized samples were evaluated through dynamic breakthrough experiments with highly volatile hexamethyldisiloxane (L2) as a model pollutant. Based on SEM, TEM, XRD, BET, FTIR, and Raman characterization, the relationship between the structure and adsorption properties of the Fe_3_O_4_–urea–rGOA was revealed, and the mechanism of the self-assembly of the graphene sheets/Fe_3_O_4_ induced by Fe^3+^ was clarified. Furthermore, the influential factors on the adsorption capacity were investigated, and adsorption–desorption tests were also carried out on the best Fe_3_O_4_–urea–rGOA adsorbent. The fabricated Fe_3_O_4_–urea–rGOAs showed high hydrophobicity and textural properties, indicating that they have great potential for VMS removal.

## 2. Results and Discussion

### 2.1. Effects of Modifier Fe(NO_3_)_3_·9H_2_O on Texture Properties

**Figure 1:**
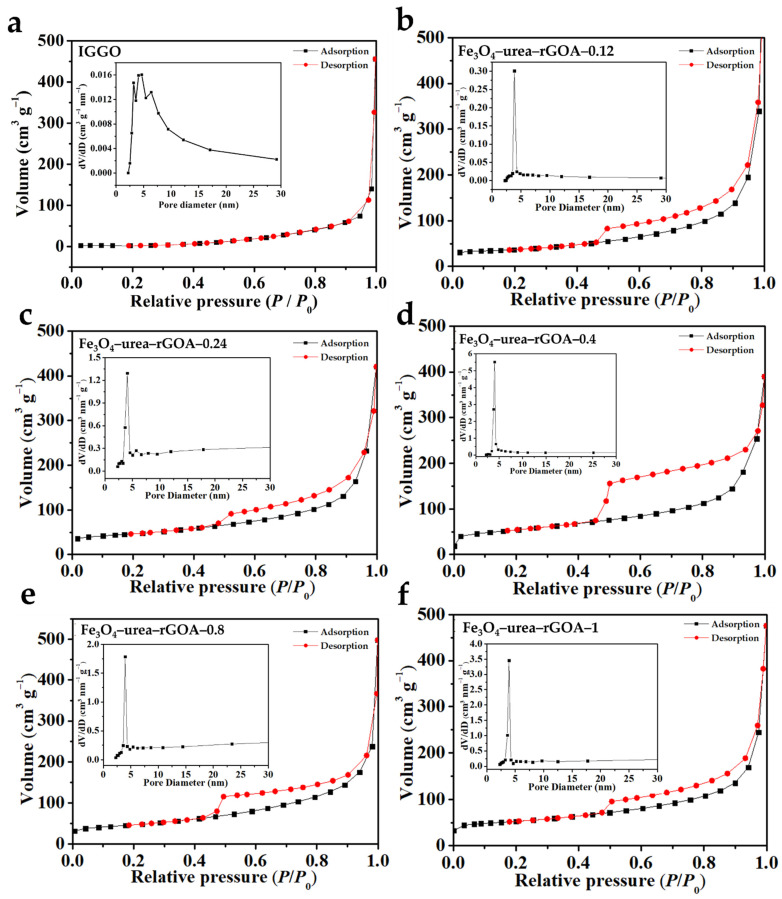
N_2_ adsorption–desorption isotherm (77 K) and the corresponding pore size distribution (inset) of IGGO (**a**), Fe_3_O_4_–urea–rGOA–0.12 (**b**), Fe_3_O_4_–urea–rGOA–0.24 (**c**), Fe_3_O_4_–urea–rGOA–0.4 (**d**), Fe_3_O_4_–urea–rGOA–0.8 (**e**), and Fe_3_O_4_–urea–rGOA–1 (**f**).

Digital photos of the Fe_3_O_4_–urea–reduced graphene oxide hydrogels (Fe_3_O_4_–urea–rGOHs) are shown in Figure S1. It can be seen that the Fe_3_O_4_–urea–rGOHs all show an overall macroscopic shape, which suggests that, under the action of reduction-induced self-assembly, the formation of hydrogels was promoted [38]. The N_2_ adsorption–desorption isotherms of IGGO and the Fe_3_O_4_–urea–rGOAs are shown in Figure 1. According to the IUPAC classification method [39], the adsorption and desorption isotherms of N_2_ on the six samples were type I and type IV, respectively, and the adsorption of the monolayer in the low-relative-pressure region reflected the phenomenon of micropore filling. With an increase in the relative pressure, multilayer adsorption appeared, and adsorption hysteresis appeared above a relative pressure of 0.4, reflecting mesoporous capillary condensation. Moreover, from a shape analysis of the hysteresis loops, the Fe_3_O_4_–urea–rGOAs were H3-type hysteresis loops, and the shapes of their holes were slit and crack holes, which were speculated to be formed due to the collaborative self-assembly process of Fe_3_O_4_ and graphene sheets [26]. According to IUPAC rules, IGGO showed type III isotherms and did not have hysteresis loops, from which it can be inferred that IGGO has a relatively non-porous/macroporous structure. As shown in Table 1, the pore structure parameters of IGGO and the Fe_3_O_4_–urea–rGOAs are listed. The BET specific surface areas of the IGGO, Fe_3_O_4_–urea–rGOA–0.12, Fe_3_O_4_–urea–rGOA–0.24, Fe_3_O_4_–urea–rGOA–0.4, Fe_3_O_4_–urea–rGOA–0.8, and Fe_3_O_4_–urea–rGOA–1 samples were found to be 7, 124, 160, 188, 177, and 162 m^2^ g^−1^, respectively. With an increase in Fe^3+^ loading, Fe_3_O_4_–urea–rGOA provided a large number of effective channels, making the BET specific surface area and total pore volume gradually increase, reaching 188 m^2^ g^−1^ and 0.77 cm^3^ g^−1^, respectively; however, with a continuous increase in Fe^3+^, the number of micropores in the complex Fe_3_O_4_–urea–rGOAs reduced, resulting in a decrease in the BET specific surface area and total pore volume [40,41]. In addition, it can be determined from the pore size distribution that the pore size was mainly in the range from 2 to 5 nm, which is a micro/narrow mesoporous range that is most suitable for L2 removal and is the main contribution to increasing the specific surface area.

### 2.2. Characterization of Adsorbents

**Figure 2:**
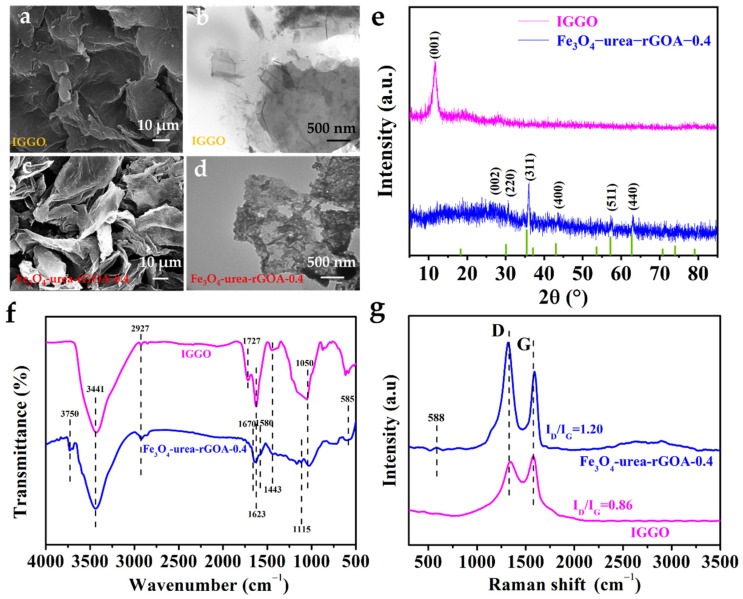
SEM and TEM of IGGO (**a**,**b**), SEM and TEM of Fe_3_O_4_–urea–rGOA–0.4 (**c**,**d**), XRD patterns of IGGO and Fe_3_O_4_–urea–rGOA–0.4 (**e**), FTIR spectra of IGGO and Fe_3_O_4_–urea–rGOA–0.4 (**f**), and Raman spectra of IGGO and Fe_3_O_4_–urea–rGOA–0.4 (**g**).

Figure 2a–d show the SEM and TEM images of IGGO and Fe_3_O_4_–urea–rGOA–0.4. Compared to IGGO, Fe_3_O_4_–urea–rGOA–0.4 exhibited an abundant three–dimensional network structure and more fissured pores. Moreover, it can be seen that the Fe_3_O_4_ spherical small particles were evenly distributed in the graphene lamellar structure, which may have increased the roughness of the graphene aerogel surface and thus enlarged the specific surface area of the graphene aerogel [40]. The XRD testing results of IGGO and Fe_3_O_4_–urea–rGOA–0.4 are shown in Figure 2e. After hydrothermal reduction, the graphene (002) diffraction peak can be seen at 24.5°, and the diffraction peaks of Fe_3_O_4_–urea–rGOA–0.4 appeared at 30.6°, 35.9°, 43.4°, 52.1°, 57.3°, and 62.9°, corresponding to the crystal planes (200), (311), (400), (422), (511), and (400) of Fe_3_O_4_, respectively. This being the case, the above-mentioned results suggest that the Fe_3_O_4_ nanoparticles were successfully combined with graphene sheets [42]. The FTIR spectrum of IGGO showed a number of absorption peaks (Figure 2f). The significant broad peaks were located at 3441 cm^−1^ for the stretching vibration of O–H in the adsorbed–state H_2_O, C–OH groups, and –COOH groups [43]. The absorption peak at 2927 cm^−1^ was attributed to the –C–H stretching vibration, [44] and that at 1727 cm^−1^ was due to the C=O stretching vibration in carboxyl. The peaks appearing at 1443 and 1623 cm^−1^ were attributed to the C=C vibration of the graphene skeleton [45]. There was a strong absorption peak at 1050 cm^−1^, which belonged to the stretching vibration of the epoxy group C–O–C [45]. After hydrothermal reduction, in the FTIR spectrum of Fe_3_O_4_–rGOA–0.4, the disappearance of the 1727 cm^−1^ peak and the appearance of the 3750 cm^−1^ peak were partly due to the possible decarboxylation reaction of the carboxyl group at 800 °C and partly due to C=O being reduced to C–OH moieties that were free [24]. The C=C stretching frequency intensity at 1623 cm^−1^ was decreased and the C–O–C stretching frequency intensity at 1050 cm^−1^ was weakened. Moreover, two new peaks that appeared at 1580 and 1115 cm^−1^ were attributed to the C=N and C–N–C stretching vibrations, respectively [46]. The appearance of another peak in the region of 1670 cm^−1^ can be attributed to the formation of an amide bond via the reaction of urea with the –COOH groups [47]. The above results reveal that the oxygen–containing groups were reduced, epoxy rings were opened via the addition of –NH–CO–NH_2_, with subsequent tautomerization to –N=C–OH–NH_2_, and that some N atoms were doped into the graphene sheet [24,46]. Furthermore, the shoulder between 1580 cm^−1^ and 1623 cm^−1^, as well as the band at 585 cm^−1^, could be due to the Fe–O stretching vibration in Fe_3_O_4_, indicating that Fe_3_O_4_ was successfully anchored on the graphene sheet, which is consistent with the XRD results [42]. In Figure 2f, the Raman spectra of the IGGO and Fe_3_O_4_–rGOA–0.4 samples are given. The ratio of the intensities of the D and G peaks (*I*_D_/*I*_G_) was in the order of IGGO (0.86) < Fe_3_O_4_–rGOA–0.4 (1.20), indicating that the defects of Fe_3_O_4_–rGOA–0.4 were relatively high, which is presumed to be due to a large number of sp^3^ hybrid carbon atom defects caused by the urea hydrothermal reduction process [45]. Moreover, a small peak at 588 cm^−1^ could be due to the Raman spectrum of Fe_3_O_4_ [27]. This indicates that Fe_3_O_4_ and rGOA were successfully combined, which is consistent with the results of previous studies.

**Figure 3:**
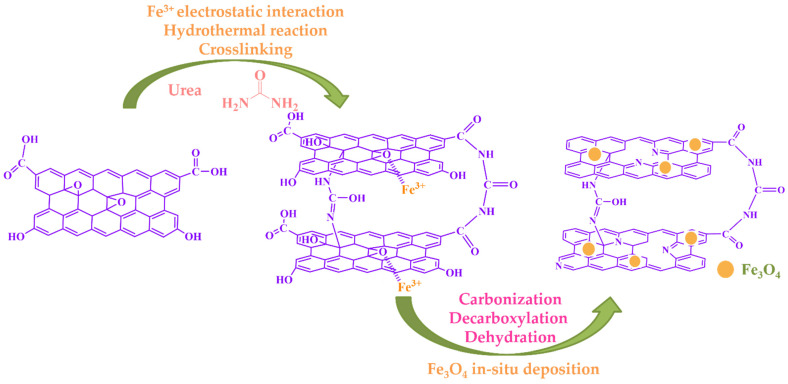
Proposed mechanism of urea reduction, crosslinking, and Fe_3_O_4_ deposition.

According to the above experimental results, we propose a possible working mechanism, as shown in Figure 3. Based on the synergic self-assembly effect induced by Fe^3+^, the two assembly processes of Fe_3_O_4_ and urea reduction were carried out simultaneously and promoted each other. The Fe_3_O_4_ nanoparticles were successfully anchored on the layer of reduced graphene oxide. Meanwhile, the N atoms were successfully doped into the graphene structure, resulting in defects on the graphene sheets, and urea also acted as a crosslinker and reducing agent. According to the literature [48,49], all of the above synergies are beneficial to the formation of a large specific surface area and micro/narrow mesoporous pores, which are the most suitable for L2 removal.

### 2.3. Comparison of Dynamic Adsorption Performances

**Figure 4:**
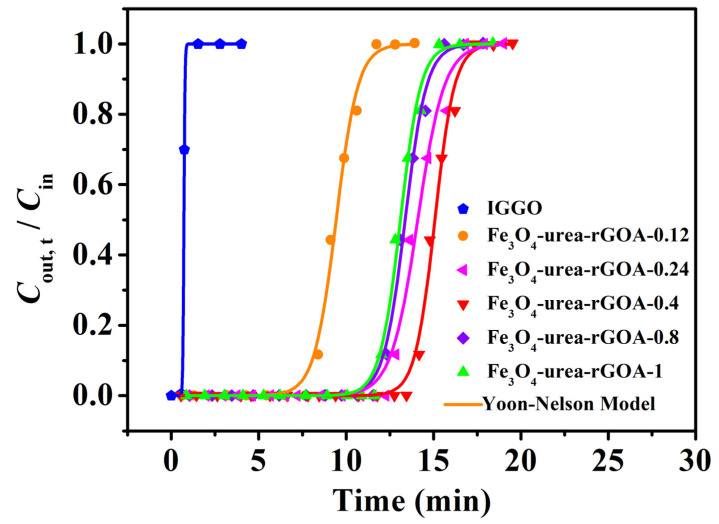
Breakthrough adsorption fitted curves of the IGGO and Fe_3_O_4_–urea–rGOAs for L2.

**Figure 5:**
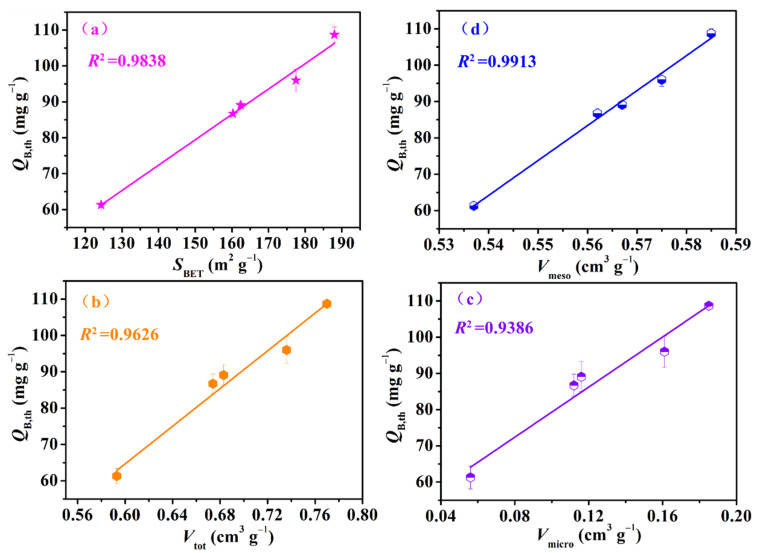
Relationship curves between *Q*_B,th_–*S*_BET_ (**a**), *Q*_B,th_–*V*_meso_ (**b**), *Q*_B,th_–*V*_tot_ (**c**), and *Q*_B,th_–*V*_micro_ (**d**) for the Fe_3_O_4_–urea–rGOAs.

The breakthrough adsorption curves (represented by the *C*_out,t_/*C*_in_~*t* relationship curve), obtained by using the Yoon–Nelson model for IGGO and Fe_3_O_4_–urea–rGOAs, are shown in Figure 4. The experimental conditions were as follows: *T* = 25 °C, *C*_in_ = 14.62 mg L^−1^, and *V*_g_ = 50 mL min^−1^. The calculated model parameters and adsorption experiment results are summarized in Table 2. The results demonstrated the following: (1) The L2 breakthrough curves for IGGO and the Fe_3_O_4_–urea–rGOAs were S–shaped, and experienced three stages of plateau–penetration–equilibrium with an extension in time, which is in line with the typical characteristics of gas–solid adsorption behavior. (2) These dynamic adsorption data can be well described with the Yoon–Nelson model equation (correlation coefficient *R*^2^ > 0.99). Therefore, in our later discussions, we chose the theoretical parameter values (*t*_B,th_, *Q*_B,th_, and *Q*_m,th_) via this model to analyze the adsorption performances of the Fe_3_O_4_–urea–rGOAs. (3) With an increase in the mass of Fe(NO_3_)_3_·9H_2_O, the adsorption performance of the Fe_3_O_4_–urea–rGOAs to L2 first increased and then decreased. The order of the adsorption properties of the Fe_3_O_4_–urea–rGOAs for L2 was as follows: Fe_3_O_4_–urea–rGOA–0.4 > Fe_3_O_4_–urea–rGOA–0.24 > Fe_3_O_4_–urea–rGOA–0.8 > Fe_3_O_4_–urea–rGOA–1 > Fe_3_O_4_–urea–rGOA–0.12 > IGGO. Among them, the *t*_B,th_, *Q*_B,th_, and *Q*_m,th_ values of Fe_3_O_4_–urea–rGOA–0.4 were 13.88 min, 101.5 mg g^−1^, and 112.4 mg g^−1^, respectively, exhibiting the best adsorption capacity of L2. (4) In order to reveal the structure–activity relationship between the texture and adsorption properties of the Fe_3_O_4_–urea–rGOAs, correlational analyses for *Q*_B,th_ with each of the *S*_BET_, *V*_meso_, *V*_tot_, and *V*_micro_ separately were performed for the Fe_3_O_4_–urea–rGOAs, and a linear simulation was performed using the y = a + bx equation, as shown in Figure 5a–d. The relevant parameters simulated by the equation are shown in Table 3. The *R*^2^ of the *Q*_B,th_–*S*_BET_, *Q*_B,th_–*V*_meso_, *Q*_B,th_–*V*_tot_, and *Q*_B,th_–*V*_micro_ linear fitting were 0.9838, 0.9913, 0.9626, and 0.9386, respectively. Therefore, the specific surface area and pore volume were the main influencing factors of L2 adsorption, and, moreover, the correlation between *V*_meso_ and *Q*_B,th_ was slightly greater than that of *V*_micro_. It has been reported that the molecular kinetic diameter of L2 is 1.044 nm [17], and when the pore size is two to four times that of the adsorbent molecule diameter, it is favorable for adsorption [17]. According to the experimental results, the prepared aerogels had a pore size ranging from 2 nm to 5 nm, which is the most suitable pore structure for absorbing L2. Therefore, the adsorption of L2 is mainly microporous and mesoporous. It can thus be inferred that capillary condensation and micropore filling were the main adsorption mechanisms of L2 on the Fe_3_O_4_–urea–rGOA–0.4 [14,24,41], and the schematic diagram of the adsorption mechanism is as shown in Figure S2.

### 2.4. Effect of Process Conditions on Adsorption

**Figure 6:**
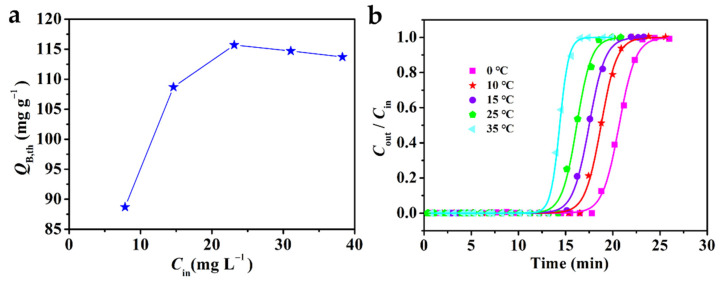
The relationship between adsorption capacity and L2 inlet concentration (**a**) and the breakthrough curves for Fe_3_O_4_–urea–rGOA–0.4 by Yoon–Nelson model (**b**).

There were many factors affecting the adsorption process [50,51]. Therefore, it is interesting to explore the influence of different Fe_3_O_4_–urea–rGOA–0.4 bed temperatures (T) and L2 inlet concentrations (C_in_) on the adsorption. The change curve of Q_B,th_ with C_in_ is shown in Figure 6a. In a low-concentration range, Q_B,th_ increased with a greater C_in_. When C_in_ reached a certain concentration, Q_B,th_ maintained equilibrium. It has been reported that when the adsorption force of an L2 molecule and the coverage of an adsorption site reach equilibrium, the adsorption capacity reaches a stable value under the corresponding conditions [52,53]. Therefore, it is appropriate to control the inlet concentration of L2 in the range from 25 mg L^−1^ to 40 mg L^−1^. Figure 6b shows the breakthrough curve of the adsorption of L2 with Fe_3_O_4_–urea–rGOA–0.4 fitted by the Yoon–Nelson model at various bed temperatures (0–55 °C). In addition, the calculated model parameters and theoretical metrics (t_B,th_, Q_B,th_, and Q_m,th_) based on the experimental data are listed in Table 4. When C_in_ was 14.62 mg L^−1^ and V_g_ was 50 mL min^−1^ at 0 °C, the maximum penetration adsorption capacity of L2 on Fe_3_O_4_–urea–rGOA–0.4 was 146.5 mg g^−1^. It can be seen that, with an increase in bed temperature, t_B,th_, Q_B,th_, and Q_m,th_ all decreased, indicating that the L2 adsorption process in the Fe_3_O_4_–urea–rGOA–0.4-filled bed was exothermic, which could further support the idea that the primary adsorption mechanisms of L2 are capillary condensation and micropore filling [14,24,41].

### 2.5. Recycling Performance of Fe_3_O_4_–Urea–rGOA–0.4

**Figure 7:**
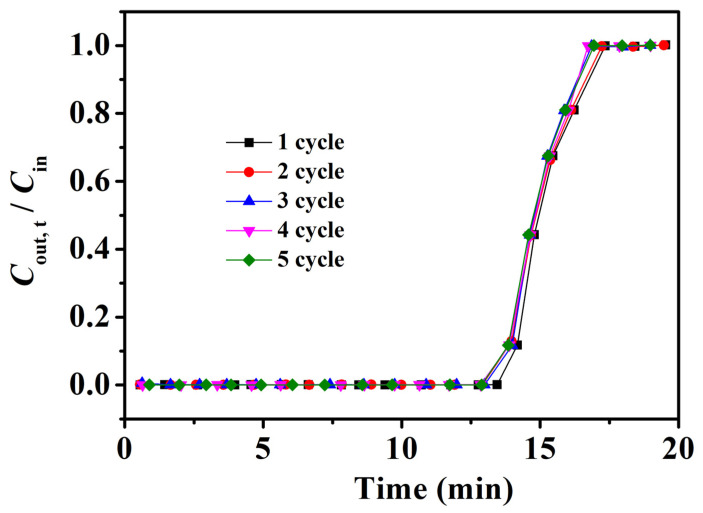
Breakthrough curves for L2 of Fe_3_O_4_–urea–rGOA–0.4 in five cycles of adsorption/desorption.

The recovery performance of adsorbents is an important factor with which to evaluate their practical application. Therefore, Fe_3_O_4_–urea–rGOA–0.4, after the adsorption of L2, was regenerated after being treated in an 80 °C water bath for 30 min, and repeated for five adsorption–desorption cycles. Thus, the experimental results of these five cycles are shown in Figure 7. As can be shown, the adsorption breakthrough curves of Fe_3_O_4_–urea–rGOA–0.4 on L2 basically coincided after five cycles of adsorption/regeneration treatment, indicating that Fe_3_O_4_–urea–rGOA–0.4 had a good recycling performance, which has prospects for industrial application. As can be seen from Table S1, although the adsorption properties were slightly lower than those of other porous carbon materials [41,54,55,56], Fe_3_O_4_–urea–rGOA–0.4 could be regenerated under a normal pressure and lower heating temperature with a more than 99% regeneration efficiency.

## 3. Materials and Methods

### 3.1. Materials and Chemicals

IGGO powder in the range from 10 to 50 μm was purchased from Suzhou Hengqiu Technology Co. (Suzhou, China). Fe(NO_3_)_3_·9H_2_O (analytical grade, Aladdin Industrial Corporation, Shanghai, China), urea (CH_4_N_2_O, analytical grade, Tianjin Yongda Chemical Reagent Co., Ltd., Tianjin, China), and deionized (DI) water were used for the preparation of the Fe_3_O_4_–urea–rGOAs. Hydrochloric acid (HCl, 99%) was purchased from Beijing Chemical Reagent Co. (Beijing, China). Hexamethyldisiloxane (L2; 99%, Aladdin Co., Ltd., Shanghai, China) was used as a representative model polluting gas of siloxane impurities in biogas.

### 3.2. Preparation of Fe_3_O_4_–Urea–rGOAs

**Figure 8:**
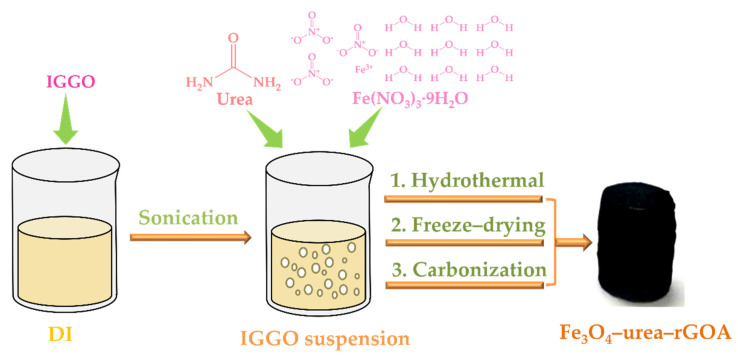
Schematic diagram of the preparation process of Fe_3_O_4_–urea–rGOA.

The schematic construction procedures of the Fe_3_O_4_–urea–rGOAs are described in Figure 8. The method used to acquire the Fe_3_O_4_–urea–rGOAs was generally divided into four steps, as described below:

Step 1: The preparation of IGGO dispersion. An amount of 0.24 g of IGGO was dispersed in 60 mL of deionized water via ultrasonic treatment for 30 min to obtain an IGGO suspension (4 mg mL^−1^).

Step 2: Hydrogel preparation. The dispersion solution was added to with a certain mass of Fe(NO_3_)_3_·9H_2_O and 0.20 g of urea (molar ratios of 0.09, 0.18, 0.30, 0.60, and 0.74), and then ultrasonic treatment was performed. After 60 min of ultrasound, the dispersion solution was transferred to a 100 mL reactor for a hydrothermal reaction at 180 °C for 8 h, acquiring the Fe_3_O_4_–urea-modified reduced graphene oxide hydrogel.

Step 3: Lyophilization and carbonization. The Fe_3_O_4_–urea–rGOHs were immersed in a solvent of ethanol for 12 h. Next, the Fe_3_O_4_–urea–rGOHs were cooled at −18 °C for 12 h, and then freeze–dried at −46 °C for 24 h. Finally, the Fe_3_O_4_–urea–rGOA sample was achieved via heating in a tube furnace at 800 °C in a N_2_ atmosphere for 2 h.

Step 4: Concentration adjustment. The same method was used to synthesize five different IGGO/Fe(NO_3_)_3_·9H_2_O mass ratios (1:0.12, 1:0.24, 1:0.4, 1:0.8, and 1:1) and denoted as Fe_3_O_4_–urea–rGOA–0.12, Fe_3_O_4_–urea–rGOA–0.24, Fe_3_O_4_–urea–rGOA–0.4, Fe_3_O_4_–urea–rGOA–0.8, and Fe_3_O_4_–urea–rGOA–1.

### 3.3. Adsorption Experiments of L2 Gas

The removal performances of IGGO and the Fe_3_O_4_–urea–rGOAs for an L2 gas stream were measured using a fixed-bed dynamic adsorption setup. The full experimental setup and related details of the methods are described in the published literature [24,25]. For each test, 0.10 g of Fe_3_O_4_–urea–rGOA was used and the experimental parameters were *T* = 25 °C, an L2 inlet concentration of 14.62 mg L^−1^, and *V*_g_ = 50 mL min^−1^. The continuous adsorption of L2 on the IGGO and Fe_3_O_4_–urea–rGOA packed-bed column was studied in terms of the breakthrough curves, which were expressed by plotting *C*_out,t_/*C*_in_ vs. adsorption time. We employed the following three metrics to evaluate the adsorbent performance: the breakthrough time (*t*_B_, defined as the time when *C*_out,t_/*C*_in_ ≈ 0.05, min); the breakthrough adsorption capacity (*Q*_B_, representing the adsorption capacity at time *t*_B_, mg g^−1^); and the saturated adsorption capacity (*Q*_m_, defined as the adsorption capacity when *C*_out, t_/*C*_in_ ≈ 1, mg g^−1^). The *Q*_B_ and *Q*_m_ values for an independent adsorption test were found using Equation (1):

(1) $$Q_{t}=\frac{V_{g}C_{\mathrm{in}}}{m}\int_{0}^{t}(1–\frac{C_{\mathrm{out},\;t}}{C_{\mathrm{in}}})dt$$

In addition to the physical quantities mentioned above, *m* is the mass of the adsorbent (g) and *V*_g_ is the flow rate of the L2 gas (L min^−1^).

### 3.4. Model for the Breakthrough Curves

The measured dynamic data of the L2 gas can be predicted via the Yoon–Nelson model, which is a semi-empirical model. The Yoon–Nelson model is represented via Equation (2):

(2) $$\frac{C_{\mathrm{out},\;t}}{C_{\mathrm{in}}}=\frac{1}{1+\exp\left[K_{\mathrm{YN}}\left(\tau –t\right)\right]}\times 100$$

where *K*_YN_ is the Yoon–Nelson constant and *τ* is the time required for retaining 50% of the initial adsorbate.

### 3.5. Regeneration of the Spent Fe_3_O_4_–Urea–rGOAs

When the adsorption of the adsorbents was saturated, it was necessary to regenerate it to achieve the cyclic adsorption of the spent Fe_3_O_4_–urea–rGOA. The adsorption tubes were placed in a water bath (80 °C) and blown with 100 mL min^−1^ of N_2_ for 30 min. Five consecutive adsorption/desorption cycles were repeated in the same manner.

### 3.6. Characterization

The SEM images were performed using a field emission scanning electron microscope (SEM, Hitachi S4800, Chiyoda City, Japan) at an accelerating voltage of 15 kV. Photos were taken of different samples using a field emission transmission electron microscope (TEM, H-7650, Hitachi, Tokyo, Japan) at 150 kV. The structures were characterized by using a D8 Advance X-ray diffractometer equipped with Cu Kα radiation (XRD, *λ* = 0.154 nm, Bruker, Bremen, Germany). FTIR spectroscopy was performed by using an FTIR spectrometer (IR Tracer–100, Shimadzu, Nagoya, Japan) in the region of 4000~500 cm^−1^. The Raman spectroscopy measurements were carried out using a Raman spectrometer (XploRA PLUS, Horiba, Japan) with a 514 nm laser. The nitrogen (N_2_) adsorption–desorption isotherms of IGGO and the Fe_3_O_4_–urea–rGOAs were collected at 77 K on a Kubo × 1000 surface area and pore size analyzer (Beijing Builder, Beijing, China). The BET surface area (*S*_BET_) was determined through the Brunauer–Emmett–Teller (BET) theory, and the pore volumes were processed through Barrett–Joyner–Halenda (BJH) models. The concentration of L2 in the gas stream was analyzed using a Fuli Analytical Instrument 9790 gas chromatograph equipped with a flame ionization detector (GC–FID, Chengde, China).

## 4. Conclusions

A series of Fe_3_O_4_–urea–rGOAs was prepared by controlling the amount of Fe_3_O_4_ precursor and urea, through which the dynamic adsorption behaviors of the hexamethyldisiloxane (L2) impurity gas in biogas were investigated. The synergistic effect between the rich porous structure of Fe_3_O_4_ and the interconnected three-dimensional pores of the rGOA greatly enlarged the specific surface area and pore volume of the rGOA. Thus, Fe_3_O_4_–urea–rGOA–0.4 exhibited the highest *S*_BET_ (188 m^2^ g^−1^), *V*_micro_ (0.19 cm^3^ g^−1^), *V*_meso_ (0.58 cm^3^ g^−1^), and *V*_tot_ (0.77 cm^3^ g^−1^). *Q*_B,th_ showed an excellent linear relationship with *S*_BET_ (*R*^2^ = 0.9838) and *V*_meso_ (*R*^2^ = 0.9931), indicating that both *S*_BET_ and *V*_meso_ were important parameters influencing the adsorption of L2 and that the main adsorption mechanisms were capillary condensation and micropore filling. Moreover, it turned out that a lower temperature and higher inlet concentration could improve the siloxane adsorption level of Fe_3_O_4_–urea–rGOA–0.4. Regeneration could be achieved by heating in a water bath at 80 ℃ for 30 min, and after five cycles, the recycling efficiency was 99%. As expected, a high adsorption capacity and excellent cycling properties made Fe_3_O_4_–urea–rGOA–0.4 a promising adsorbent for VMS removal in industrial applications.

## Figures and Tables

**Table 1:** Texture properties of samples.

Samples	*S*_BET_ (m^2^ g^−1^)	*V*_tot_ (cm^3^ g^−1^)	*V*_micro_ (cm^3^ g^−1^)	*V*_meso_ (cm^3^ g^−1^) ^a^	*D*_aver_ (nm)
IGGO	7	0.23	0.01	0.22	13.18
Fe_3_O_4_–urea–rGOA–0.12	124	0.59	0.06	0.53	5.02
Fe_3_O_4_–urea–rGOA–0.24	160	0.67	0.11	0.56	4.95
Fe_3_O_4_–urea–rGOA–0.4	188	0.77	0.19	0.58	3.94
Fe_3_O_4_–urea–rGOA–0.8	178	0.74	0.16	0.58	4.88
Fe_3_O_4_–urea–rGOA–1	162	0.68	0.12	0.56	4.95

^a^ *V*_meso_ = *V*_tot_ − *V*_micro_.

**Table 2:** Adsorption parameters of IGGO and Fe_3_O_4_–urea–rGOAs for L2.

Adsorbents	Experimental			Model					
	*t*_B_/ min	*Q*_B_/ mg g^−1^	*Q*_m_/ mg g^−1^	*t*_B,th_/ min	*Q*_B,th_/ mg g^−1^	*Q*_m,th_/ mg g^−1^	*K_YN_*	*τ*/ min	*R* ^2^
IGGO	0.53	3.9	5.8	0.65	4.8	5.2	34.06	0.72	0.9999
Fe_3_O_4_–urea–rGOA–0.12	8.00	58.4	69.4	7.60	55.6	68.7	1.59	9.42	0.9945
Fe_3_O_4_–urea–rGOA–0.24	11.94	87.2	98.2	11.60	84.8	97.7	1.63	13.38	0.9962
Fe_3_O_4_–urea–rGOA–0.4	13.77	100.5	110.9	13.88	101.5	112.4	1.77	15.07	0.9945
Fe_3_O_4_–urea–rGOA–0.8	12.48	91.2	103.8	11.86	86.7	102.7	1.31	14.10	0.9906
Fe_3_O_4_–urea–rGOA–1	11.73	85.7	96.3	11.48	83.9	95.4	1.70	13.10	0.9946

**Table 3:** The statistical parameters of curves between *Q*_B,th_–*S*_BET_, *Q*_B,th_–*V*_meso_, *Q*_B,th_–*V*_tot_, and *Q*_B,th_–*V*_micro_ for the Fe_3_O_4_–urea–rGOAs.

Statistical Parameters		*Q*_B,th_–*S*_BET_	*Q*_B,th_–*V*_meso_	*Q*_B,th_–*V*_tot_	*Q*_B,th_–*V*_micro_
Number of Points		5	5	5	5
Equation		y = a + bx			
Residual Sum of Squares		14.6870	7.8656	33.8747	55.5914
Standard Deviation		1.9162	1.4023	2.9101	3.7280
*R*-Square		0.9838	0.9913	0.9626	0.9386
Intercept	Value	−27.6825	−455.2833	−87.0625	45.3823
	Standard Error	7.4994	25.4242	17.2712	5.7807
Slope	Value	0.7141	961.8600	253.7942	341.0927
	Standard Error	0.04575	44.9644	24.8926	43.2595

**Table 4:** Influence of the temperature on the adsorption of L2 over Fe_3_O_4_–urea–rGOA–0.4 ^a^.

Term	Value	*t*_B,th_/ min	*Q*_B,th_/ mg g^−1^	*Q*_m,th_/ mg g^−1^	*K*_YN_	*τ*/ min	*R* ^2^
*T*/°C	0	18.08	132.2	146.5	1.1431	20.66	0.9982
	10	16.28	119.0	131.8	1.1901	18.75	0.9979
	15	14.97	109.4	121.3	1.1705	17.49	0.9989
	25	13.88	101.5	112.4	1.2814	16.18	0.9963
	35	12.95	94.7	105.0	2.0786	14.37	0.9981

^a^ C_in_ = 14.62 mg L^−1^, m = 0.10 g, and V_g_ = 50 mL min^−1^.

## Data Availability

Data is contained within the article. The data presented in this study are available.

## Supplementary Materials

The following supporting information can be downloaded at: https://www.mdpi.com/article/10.3390/molecules28186622/s1, Figure S1: Photographs of the Fe_3_O_4_–urea–rGOHs. Figure S2: The schematic diagram of the adsorption mechanism of L2 on the Fe_3_O_4_–urea–rGOAs. Table S1: Adsorption and regeneration capacities of different porous carbon materials for VMS.

## Sample Availability

Samples of the compounds are not available from the authors.
