# Supplementary material for: Hexamethyldisiloxane Removal from Biogas Using a Fe3O4–Urea-Modified Three-Dimensional Graphene Aerogel
Source: Molecules. 2023 Sep 14;28(18):6622. doi: 10.3390/molecules28186622 (PMC10535819; doi:10.3390/molecules28186622)
Supplement: Supplementary file 1 [file molecules-28-06622-s001.zip › molecules-2564927-supplementary.pdf]

Supplementary information

Hexamethyldisiloxane Removal from Biogas Using a Fe_3O_4–Urea–Modified Three–Dimensional Graphene Aerogel

Siqi Lv [1], Xifeng Hou [3], Yanhui Zheng [1,2,*] and Zichuan Ma [1,*]

[1] Hebei Key Laboratory of Inorganic Nano–Materials, College of Chemistry and Material Sciences, Hebei Normal University, Shijiazhuang 050024, China; lvsq@stu.hebtu.edu.cn (S.L.)
[2] College of Chemical Technology, Shijiazhuang University, Shijiazhuang 050035, China
[3] Hebei Key Laboratory of Animal Physiology, Biochemistry and Molecular Biology, College of Life Sciences, Hebei Normal University, Shijiazhuang, Hebei 050024, China; houxf@stu.hebtu.edu.cn (X.H.)
[*] Correspondence: zhengyh0308@stu.hebtu.edu.cn (Y.Z.); mazc@hebtu.edu.cn (Z.M.); Tel.: +86–0311–80787400 (Z.M.)

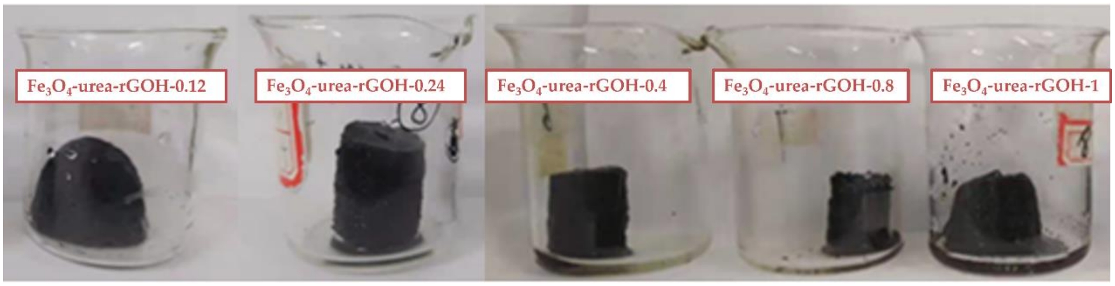

Figure S1. Photographs of the Fe_3O_4–urea–rGOHs.

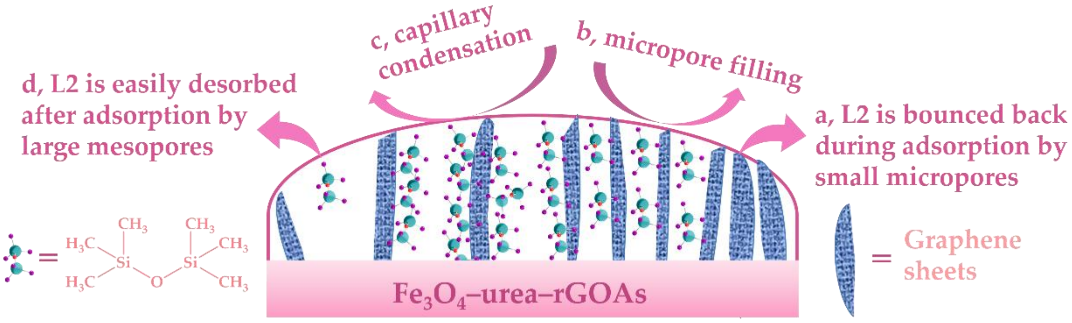

Figure S2. The schematic diagram of the adsorption mechanism of L2 on the Fe_3O_4–urea–rGOAs.

Table S1. Adsorption and regeneration capacities of different porous carbon materials for VMS.

Adsorbent	Adsorbed gas	Q_m (mg g^{-1})	Regeneration Method	RE[a] (%)	Ref.
AC	D3	60–878	Heating at 100–200 °C	50	[54]
AC	L2	10–100	Four–step heating treatment at 80–160 °C	70–80	[55]
AC	D4	526	By the oxidation with H_2O_2 and O_3	40–92	[56]
FeMBC–3	L2	356.4	Heating at 100 °C for 270 min	93	[41]
Fe_3O_4–urea–rGOA–0.4	L2	146.5	Heating at 80 °C for 30 min	> 99	This work

[a] Regeneration efficiency after 1 cycle (RE).
